# Evolutionary History and Population Dynamics of Hepatitis E Virus

**DOI:** 10.1371/journal.pone.0014376

**Published:** 2010-12-17

**Authors:** Michael A. Purdy, Yury E. Khudyakov

**Affiliations:** Division of Viral Hepatitis, Centers for Disease Control and Prevention, Atlanta, Georgia, United States of America; Saint Louis University, United States of America

## Abstract

**Background:**

Hepatitis E virus (HEV) is an enterically transmitted hepatropic virus. It segregates as four genotypes. All genotypes infect humans while only genotypes 3 and 4 also infect several animal species. It has been suggested that hepatitis E is zoonotic, but no study has analyzed the evolutionary history of HEV. We present here an analysis of the evolutionary history of HEV.

**Methods and Findings:**

The times to the most recent common ancestors for all four genotypes of HEV were calculated using BEAST to conduct a Bayesian analysis of HEV. The population dynamics for genotypes 1, 3 and 4 were analyzed using skyline plots. Bayesian analysis showed that the most recent common ancestor for modern HEV existed between 536 and 1344 years ago. The progenitor of HEV appears to have given rise to anthropotropic and enzootic forms of HEV, which evolved into genotypes 1 and 2 and genotypes 3 and 4, respectively. Population dynamics suggest that genotypes 1, 3 and 4 experienced a population expansion during the 20^th^ century. Genotype 1 has increased in infected population size ∼30–35 years ago. Genotype 3 and 4 have experienced an increase in population size starting late in the 19^th^ century until ca.1940-45, with genotype 3 having undergone additional rapid expansion until ca.1960. The effective population size for both genotype 3 and 4 rapidly declined to pre-expansion levels starting in ca.1990. Genotype 4 was further examined as Chinese and Japanese sequences, which exhibited different population dynamics, suggesting that this genotype experienced different evolutionary history in these two countries.

**Conclusions:**

HEV appears to have evolved through a series of steps, in which the ancestors of HEV may have adapted to a succession of animal hosts leading to humans. Analysis of the population dynamics of HEV suggests a substantial temporal variation in the rate of transmission among HEV genotypes in different geographic regions late in the 20^th^ Century.

## Introduction

Hepatitis E virus (HEV) is a positive-sense, single-stranded RNA virus. It is a member of the genus *hepevirus* in the *Hepeviridae* family [1]. The genome contains three open reading frames (ORFs). The 5′-most ORF, ORF1, codes for non-structural genes, while the partially overlapping ORF2 and ORF3 encode for a structural protein and a protein of unknown function, respectively [2].

HEV is the causative agent of hepatitis E. The first outbreaks of hepatitis E described in the literature were waterborne and were associated with fecally contaminated water sources [3], [4]. The disease primarily affects young to middle-aged adults, and is self-limiting but tends to lead to high mortality rates among pregnant women. Until its discovery in swine (*Sus scrofa*), hepatitis E was assumed to be limited to developing countries, and was seen only in industrialized countries as imported cases [2], [3]. However, HEV can be transmitted zoonotically to humans such as farm workers and veterinarians who work with swine [4]–[9]. More evidence from Japan and Europe suggests that HEV can be transmitted to humans from consumption of meat and offal of wild boars (which also belong to *S. scrofa*) and sika deer (*Cervus nippon*) [9]–[12].

HEV has one serotype but can be segregated into four genotypes, 1 to 4. To date, the evolutionary relationship among the four HEV genotypes in humans and swine is not completely understood. Genotypes 1 and 2 do not infect swine [13], while genotypes 3 and 4 can be found in humans and swine [9], [14]. These data suggest that HEV can be segregated into two clades. One clade is the enterically transmitted, epidemic form represented by genotypes 1 and 2, and the other is the sporadically transmitted, zoonotic form exemplified by genotypes 3 and 4 [6], [9], [13], [15]–[17]. Genotypes 1 and 2 are associated with epidemic and sporadic hepatitis E in developing countries [2], [3], while genotypes 3 and 4 are associated with sporadic disease attributable to exposure to body fluids of infected swine [8] and ingestion of food products from pigs, boars and deer [11], [16], [18].

That HEV has an animal origin [19] suggests some ancestral HEV variants could have subsequently developed capacity to efficiently transmit to and between humans. It is important to understand evolutionary and epidemiological processes facilitating this transition from enzootic to human-to-human transmission in order to prevent emergence of novel human diseases. The clear division between HEV genotypes into 2 modes of transmission offers an important opportunity for studying molecular evolutionary processes related to the transition from one mode to another. In the present work, we studied the evolutionary history of HEV using several models for estimation of the population dynamics, time to the most recent common ancestor (TMRCA), and variation in selective pressures acting on different HEV genotypes.

## Materials and Methods

### HEV Sequences

Originally fifty-five HEV sequences with known collection dates were obtained from GenBank (Table S1). There were 43 ORF1 sequences, 48 ORF2 sequences and 54 ORF3 sequences. Each ORF sequence data set (Table S2) was modified as follows. The polyproline region was removed from ORF1 in all sequences. A preliminary examination of this region across all four genotypes revealed that the polyproline region is not a hypervariable region, but rather a genotypically diverse sequence. Within each genotype, this region could be aligned unambiguously with a high degree of specificity, but alignment between genotypes was problematic. For ORF1, nucleotides 1 to 24 were removed from the sequences because sequence AY204877 was missing these bases.

vBecause ORF2 partially overlaps ORF3, ORF2 was split into two regions for further analysis: the overlap region (ORF2.O) and the non-overlap (ORF2.N) region. ORF2 sequences were segregated into overlap and non-overlap databases by splitting the ORF2 sequences between nucleotides 5446 and 5447 for genotypes 1 and 2, between nucleotides 5543 and 5544 for genotype 3 and between nucleotides 5517 and 5518 for genotype 4 (Table S2).

### Sequence Alignments

Nucleotide sequences were aligned initially using ClustalX2 (version 2.0.3) [20], amino acid sequences were aligned using MUSCLE (version 3.6; http://www.drive5.com/muscle/download3.6.html) [21], and all sequence alignments were adjusted *post hoc* by visual inspection to ensure that the alignments were biologically relevant [22].

### Selective Pressure

The ratio between nonsynonymous (*dN*) and synonymous (*dS*) substitutions, (*dN/dS*), was calculated for genotypes 1, 3 and 4 in the four selected genome regions (ORF1, ORF2.O, ORF2.N and ORF3) using HyPhy (version 0.992beta; http://www.datam0nk3y.org/hyphy/doku.php) [23] with 1-rate fixed-effects likelihood [24].

### Population Dynamics

Divergence times for the four HEV genotypes were calculated using BEAST (Version 1.4.8; http://beast.bio.ed.ac.uk/) [25]. Substitution rates for each ORF or region of the genome, used in the determination of population dynamics, were calculated according to the method of Moratorio *et al*. [26] using the original set of GenBank sequences. Dates of collection for each sequence were determined either from GenBank annotations, the journal article describing the sequence or correspondence with the submitters. Avian HEV (AY535004 and EF206691) and an HEV sequence infecting a rat (GQ504009) sequences were used as outgroup as required. The GTR substitution model with four gamma categories and invariant sites was used for each calculation. Codons were grouped into three partitions and the substitution model was unlinked across codon positions. UPGMA was used to construct a starting tree. Test runs were carried out with the HKY substitution model. The divergence times calculated with HKY were about 10% shorter than those calculated with GTR. Because of the apparent underestimation of divergence times in the analysis of some viruses [27], [28] we decided to use the more complex GTR model. Each analysis was run so that the effective sample size was greater than 200, unless otherwise noted.

Skyline plots for HEV were calculated with a variety of models without avian HEV or sequences from HEV infecting rats as outgroup. Skyline plot analysis was conducted using ORF2.N sequences. Because of the prohibitive demand on computer resources, the complete ORF1 was never used. Instead, skyline plots were constructed using the 3′-terminal 1650 nucleotides in ORF1 to confirm the results from ORF2. However, the ESS for these analyses was not run until the ESS was greater than 200, unless otherwise noted.

The Bayes factor was used for model comparison to determine which model yielded the best results. This factor was calculated for each model tested, using marginal likelihood estimated according to the method of Newton and Raftery [29], with the modifications proposed by Suchard et al. [30]. All calculations were conducted either on in-house computers or through the Cornell University Computational Biology Service Unit (http://cbsuapps.tc.cornell.edu/index.aspx). Phylogenetic trees were edited for publication using FigTree (Version 1.1.2; http://tree.bio.ed.ac.uk/software/figtree/) [31].

While the original analysis was being completed, we were provided with the dates of collection for additional sequences without such information in GenBank and new full-length HEV sequences were deposited in GenBank (Table S1). All these additional sequences were used to conduct a comprehensive analysis and to confirm the original calculations on the population dynamics.

## Results

### Genotype-specific selection-pressure

**Figure 1:**
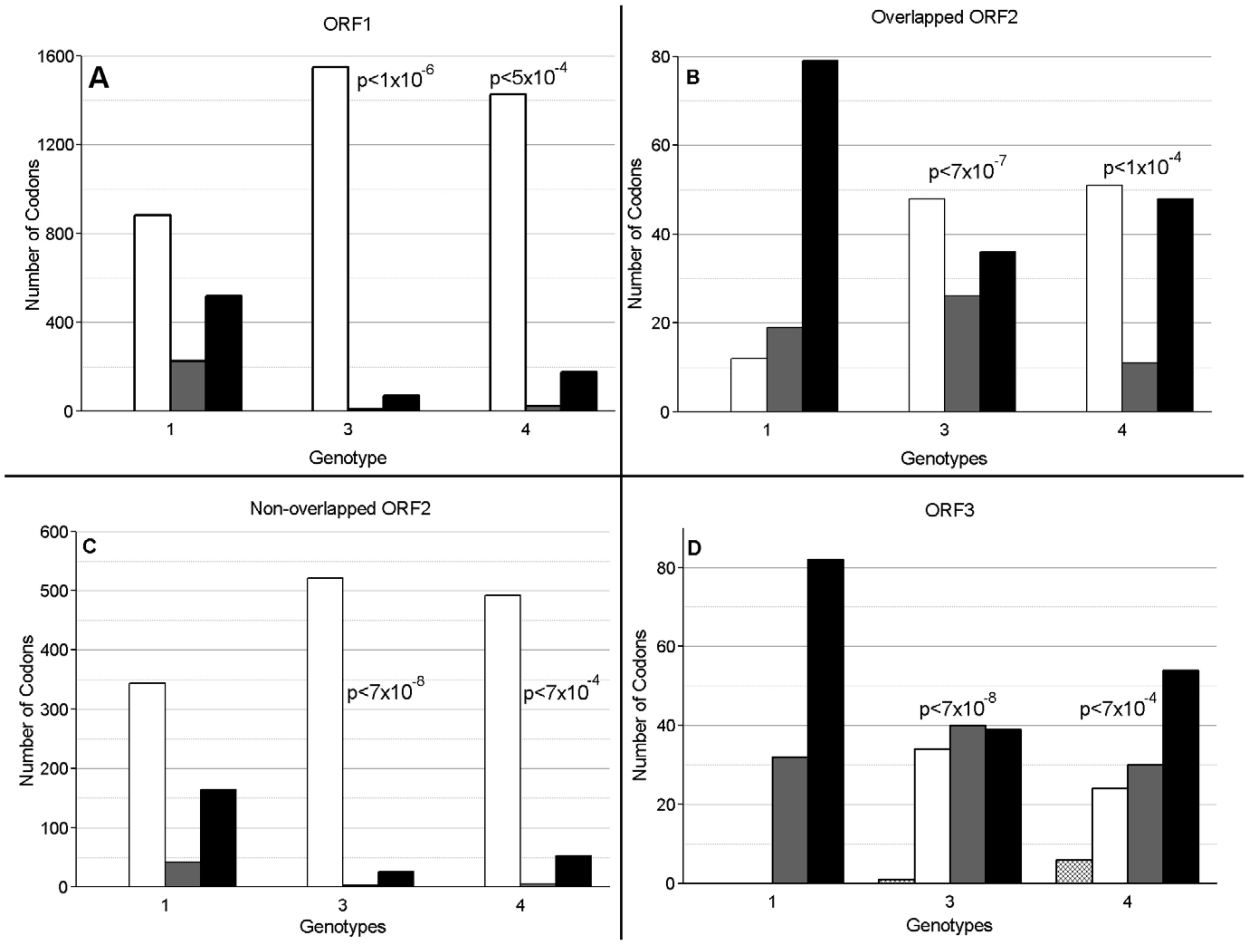
Evolutionary pressure across selected regions of the HEV genome by genotype. The regions of the HEV genome examined are A) ORF1, B) overlapped region of ORF2, C) non-overlapped region of ORF2 and D) ORF3. Selective pressure was calculated using 1 rate Fixed Effects Likelihood using HyPhy [24]. Positive selection is shown by the diagonally hatched bar, negative selection is shown by the white bar, neutral selection is shown by the gray bar and invariant sites are shown by the black bar. The chi-square p value (with Yates continuity correction) for variable versus invariant sites between genotype 1 and either genotypes 3 or 4 is shown by the values placed next to the genotype 3 and 4 data, respectively. The best chi-square value for genotype 3 against genotype 4 is p<0.15.

HEV genotypes 1 and 2 can be efficiently transmitted only among humans, whereas genotypes 3 and 4 infect swine, deer, boars and rabbits as well as humans [4], [9], [17], [18]. These two modes of transmission therefore constitute distinct phenotypes shaped under different selection pressures acting on these two groups of HEV genotypes. Analysis of selection pressures applied to protein-coding regions is frequently conducted using *dN*/*dS* ratios [23]. Considering that effective infection of many host species should require involvement of many sites for the adaptation to various genetic environments, it was expected that HEV genotypes 3 and 4 would carry more polymorphic positions than genotype 1 and 2. Indeed, HEV genotypes 3 and 4 were found to contain fewer conserved sites (p<7×10^−4^) across the entire genome than genotype 1 (Fig. 1). HEV genotype 2 was not analyzed because only one full-genome sequence (M74506) of this genotype was available.

An examination of the distribution of sites under different selection pressures (Fig. 1) showed that there were very few positively selected sites among HEV genotypes, with almost all of them located within the region of overlap between ORF3 and ORF2. However, the detection of sites with high *dN/dS* values may be an artifact of the pattern of substitution strongly affected by contrasting evolutionary constraints acting on both ORFs within the overlap region. Similarly, a high percentage of invariant sites found in this region (Fig. 1B and1D) are most probably related to the same constraints. The non-overlapped regions of the genotype 3 and 4 genomes, ORF1 and ORF2.N, are dominated by negatively selected sites (Fig. 1A and 1C). Genotype 1 has more even distribution of negative, neutral and invariant sites in ORF1 and ORF2.N than genotypes 3 and 4, but still has more negatively selected than invariant sites (Fig. 1A and 1C). This distribution of the ORF1 and ORF2.N sites clearly separates genotype 1 from genotypes 3 and 4, as would be expected for a genotype with a narrower host range.

### Time to the Most Recent Common Ancestor for HEV genotypes

In order to determine the evolutionary history of these HEV genotypes, divergence times were calculated using sequences from all four genotypes collected at well defined times. The substitution rate across the HEV genome was established using the original sequence dataset (Table S1) as described by Moratorio et al. [26]. It was found that ORF1 and ORF2.N have similar substitution rates, but ORF3 has a substitution rate 4-fold higher and the overlap region of ORF2 has a rate ∼3-fold higher than ORF1 and non-overlap region of ORF2. The ORF1 and ORF2.N rates are within the range of rates calculated by other groups (Table 1). Constraints due to the overlap between ORF3 and ORF2.O may bias the substitution rate calculations (Fig. 1B and 1D). Alternatively, this region may contain sites with frequent recurrent substitutions, reflecting a higher degree of homoplasy in this region compared to the other parts of the HEV genome, which may lead to overestimation of the substitution rate.

**Table 1:** Substitution rates calculated for HEV.

Region		Genotypes	Substitution rate	Source
ORF1		1, 2, 3 & 4	0.00130	a
ORF2				
	Overlap	1, 2, 3 & 4	0.00398	
	Non-overlap	1, 2, 3 & 4	0.00113	
ORF3		1, 2, 3 & 4	0.00557	
RNA pol		3 & 4	0.00084	b
Complete genome		4	0.00172	c
		4	0.00141	
		4	0.00140	

This table contains substitution rates calculated for several regions of the HEV genome. The column marked Region indicates the regions used for each calculation. Genotypes refers to the genotypes of sequences were used in each calculation. The next column lists the calculated substitution rates. The sources for these calculations were a) this paper, b) Tanaka et al. [19] and c) Takahashi et al. [42].

The substitution rate calculated for each region was used in BEAST to calculate divergence times for the four HEV genotypes. When the rates for ORF3 and ORF2.O were used to calculate divergence times for the most common recent ancestors (TMRCAs) for these regions, the TMRCA for all genotypes was between 15 and 32 years ago. These dates fall within the collection times for the specimens used in the calculations and may result from an inability by BEAST to accurately estimate TMRCA values from sequences with high fractional invariance and significant convergence in remaining variable sites. For this reason, ORF3 and ORF2.O were excluded from further calculations.

Using an expanded set of sequences from GenBank (Table S1) the TMRCAs for all HEV genotypes were calculated for individual genotypes 1, 3 and 4 as well as for the genotypes 1 and 2 clade, the genotypes 3 and 4 clade and the four-genotypes clade. The mean TMRCA and the 95% highest posterior probability density for these clades as calculated with a coalescent constant size tree prior and a strict clock are shown in Table 2 (values for all models tested can be found in Table S3). The Bayes factors for these nine models showed that they were all equally likely. The data from ORF2.N suggest that the mean time of emergence of the ancestor for modern HEV genotypes ranged from 536 to 1344 years ago; for genotypes 1/2, from 367 to 656 years ago; for genotypes 3/4 it was from 417 to 679 years ago; for genotype 3, from 265 to 342 years ago; for genotype 4, from 131 to 266 years ago; and for genotype 1, from 87 to 199 years ago Thus, the anthropotropic genotype 1 is the most recent compared to the enzootic genotypes 3 and 4, with all genotype 1 modern lineages coalescing ∼87–199 years ago. Comparing the divergence times for ORF1 versus that for ORF2.N shows the divergence times for genotypes 1, 3 and 4 are on average 48, 14 and 10 years older those calculated for ORF2.N, respectively. The ORF1 divergence times for the genotype 1/2 and 3/4 clades are 180 and 237 years older, respectively, than for ORF2.N, and the divergence time for the ancestor of all four genotypes is 339 years older for the ORF1 calculation than for ORF2.N (Table S3).

**Table 2:** Calculated TMRCA values for ORF2.N and ORF1.

A. ORF2.2			Relaxed Uncorrelated Clock			
	Strict Clock		Lognormal		Exponential	
Genotypes	Mean	HPD	Mean	HPD	Mean	HPD
1, 2, 3 & 4	536.49	462.04 – 614.08	556.20	437.69 – 686.09	864.78	434.16 – 1455.83
3 & 4	416.82	354.04 – 485.58	423.74	336.84 – 519.03		
1 & 2	457.66	375.23 – 540.64	441.44	309.47 – 588.95	367.18	154.89 – 665.78
3	264.54	233.40 – 298.70	277.11	229.66 – 326.38	421.26	213.42 – 678.09
4	130.73	119.56 – 143.00	146.82	124.01 – 171.23	240.26	107.36 – 415.90
1	93.10	83.76 – 102.52	94.23	80.53 – 110.60	146.44	68.98 – 246.24

B. ORF 1			Relaxed Uncorrelated Clock			
	Strict Clock		Lognormal		Exponential	
Genotypes	Mean	HPD	Mean	HPD	Mean	HPD
1, 2, 3 & 4	816.44	761.28 – 877.48	814.03	756.31 – 870.37	**1343.53**	**488.50 – 2412.12**
3 & 4	679.23	621.66 – 733.37	677.60	622.90 – 733.89	**614.87**	**298.80 – 983.28**
1 & 2	655.54	593.76 – 719.45	652.84	592.61 – 719.15	**498.96**	**269.33 – 960.38**
3	337.68	331.89 – 364.43	337.45	311.79 – 363.26	**431.81**	**205.00 – 699.57**
4	146.38	138.74 – 154.22	146.35	134.02 – 151.20	**266.28**	**130.39 – 455.44**
1	86.73	81.80 – 91.65	86.70	82.02 – 91.75	**198.89**	**87.24 – 408.47**

The values for the time to the most recent common ancestor were calculated using BEAST using the expanded sequence data set (See table S1). These values are calculated using a strict clock, an uncorrelated lognormal relaxed clock and an uncorrelated exponential relaxed clock with a coalescent constant size tree prior. The mean time and limits of the 95% highest posterior probability density are shown for each ancestor. The genotypes column shows which genotypes belong to each ancestor. Where no values are listed for the genotype 3 & 4 ancestor for the uncorrelated exponential relaxed clock this is because genotypes 3 and 4 do not share a common ancestor in this model. The bold text values denote models for which some variables have an ESS below 200.

The calculated root for the HEV genotype 1–4 tree for ORF1 and ORF2.N without an outgroup falls between clades of genotypes 3 and 4, and genotypes 1 and 2, segregating these clades into enzootic and anthropotropic classes. Although this result appears to reflect the transmission phenotype for genotypes 1 and 2, and genotypes 3 and 4, it should be noted that there is only a single genotype 2 sequence in this calculation and the future discovery of genotype 2 sequences might alter this result. Because of this we rooted the ORF2.N sequences were rooted using avian HEV sequences, AY535004 and EF206691. The avian sequences root the tree between clades of genotypes 3 and 4, and genotypes 1 and 2, in accord with the outcome of calculations without an outgroup. In addition, a calculation with an uncorrelated clock, a lognormal substitution rate and a constant growth prior estimates the time of divergence from the ancestor of avian and genotype 1–4 HEV sequences at about 1.36×10^6^ years ago (range 2.3×10^5^ to 2.6×10^6^ years ago) (Data not shown). The large divergence time between avian and HEV genotype 1–4 suggests that avian HEV is not a fifth HEV genotype [32]. Running an analysis where the ORF2.N sequences were rooted with an HEV sequence infecting a rat (GQ504009) further confirmed this phylogeny and yielded a divergence time for the ancestor of rat and human/swine HEV of about 7.44×10^4^ years ago (range 2.1×10^4^ to 1.4×10^5^ years ago). These two divergence times should be considered rough estimates; first because of the sampling bias where there are many more human and swine sequences than from avian and rat, and second because of the long branches for HEV sequences from avian and rat compared with the human and swine sequences.

### Demographic history of HEV genotypes

**Figure 2:**
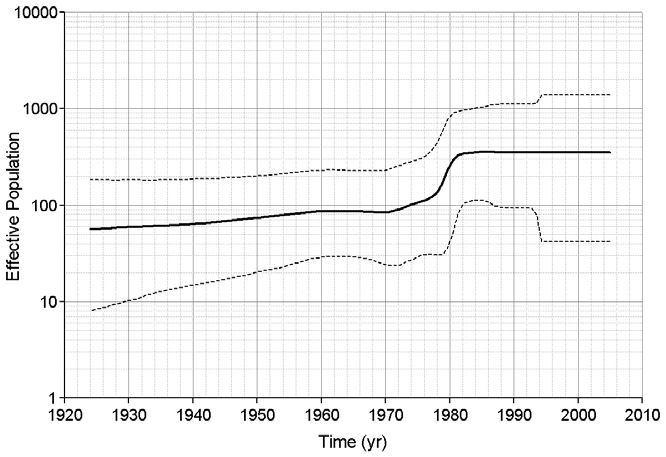
Skyline plot for genotype 1. ORF2.N genotype 1 sequences were used to construct this plot using an uncorrelated lognormal relaxed clock and a Bayesian piecewise constant skyline model with 10 groups. The solid black line represents the mean value of the skyline plot. The dashed black lines represent the limits of the 95% highest posterior probability density. Time is shown in years across the bottom of the plot and the effective population size is shown on the left-hand scale.

**Figure 3:**
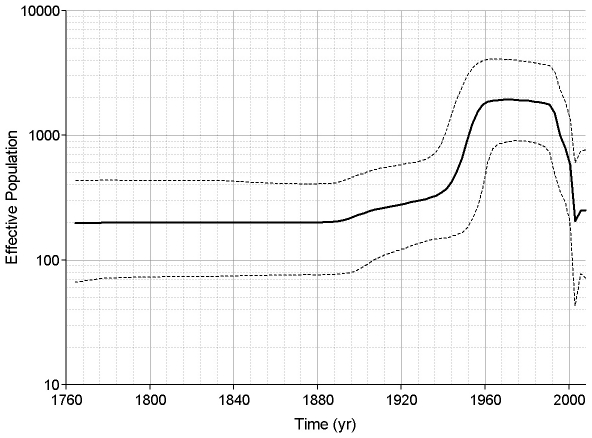
Skyline plot for genotype 3. ORF2.N genotype 3 sequences were used to construct this plot using an uncorrelated lognormal relaxed clock and a Bayesian piecewise constant skyline model with 10 groups. The scales and line designations are the same as those used in Figure 2.

**Figure 4:**
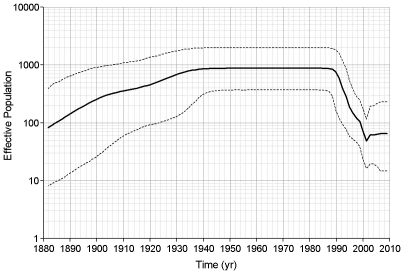
Skyline plot for genotype 4. ORF2.N genotype 4 sequences were used to construct this plot using an uncorrelated lognormal relaxed clock and a Bayesian piecewise constant skyline model with 10 groups. The scales and line designations are the same as those used in Figure 2.

Skyline plots were created to examine the population dynamics of HEV. Following Drummond et al. [33] we decided to use a relaxed lognormal clock and a piecewise constant skyline model for our skyline plots. Skyline plots using ORF2.N sequences for genotypes 1, 3 and 4 showed that each genotype is undergoing a different type of dynamics. About 25–35 years ago, genotype 1 went through an increase in population size (Fig. 2). The genotype 3 population was stable for ∼130 years from about 1760, but it has experienced a dramatic shift in its size over the 20th century, with a biphasic increase, starting approximately at the turn of the century and increasing rapidly about 1940–1960, followed by a rapid decline to the original level, starting at ∼1990 (Fig. 3). The effective population size of genotype 4 appears to have experienced a continuous expansion from 1880 to 1940. Thereafter, it remained constant until ∼20 years ago when it rapidly decreased over ∼10 years to the original level (Fig. 4). It is interesting that the time periods of expansion and decrease in population size were similar for both zoonotic genotypes. Analysis of a segment of 1650 nucleotides from the 3′ end of ORF1 resulted in concordant estimates of population dynamics for genotypes 1, 3 and 4 (data not shown).

**Figure 5:**
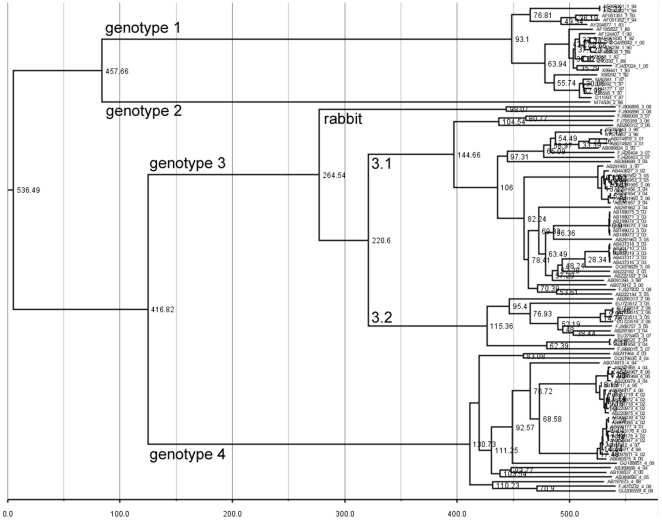
A phylogenetic tree for ORF2.N. This tree was constructed using a strict clock with a constant growth prior. The numbers at each tree node are the mean values for TMRCA at that node. The numbers across the bottom of the figure are the times in years from the calculated TMRCA for the ancestor of genotypes 1–4. Each genotype clade is labeled, and the rabbit, 3.1 and 3.2 clades are labeled within the genotype 3 clade. The genotype 3 subtypes included in each clade can be found in Table S1. Each sequence is labeled with its GenBank accession number followed by its genotype and two digit designation for its year of collection.

**Figure 6:**
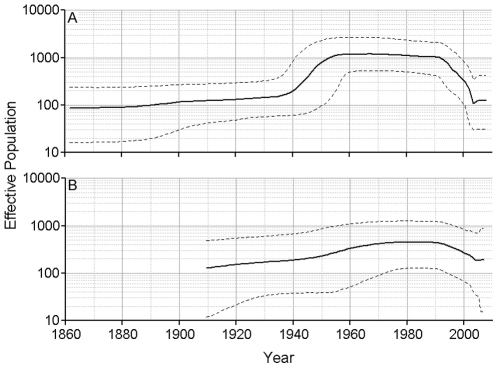
Skyline plot for genotype 3.1 and 3.2 clades. ORF2.N genotype 3 sequences without rabbit sequences were used to construct this plot using an uncorrelated lognormal relaxed clock and a Bayesian piecewise constant skyline model with 10 groups. The scales and line designations are the same as those used in Figure 2. A) clade 3.1, and B) clade 3.2. The genotype 3 subtypes included in each clade can be found in Table S1.

**Figure 7:**
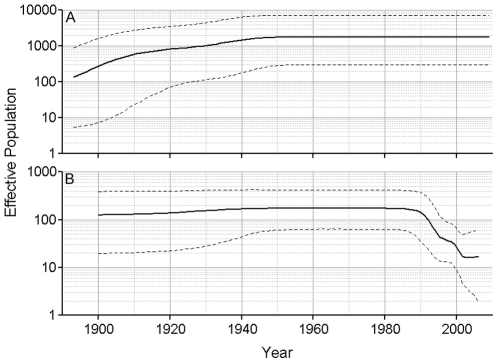
Skyline plot for genotype 4 Chinese and Japanese sequences. ORF2.N genotype 4 Chinese and Japanese sequences were used to construct this plot using an uncorrelated lognormal relaxed clock and a Bayesian piecewise constant skyline model with 10 groups for the Japanese sequences and 5 groups for the Chinese sequences. The scales and line designations are the same as those used in Figure 2. A) Chinese sequences, and B) Japanese sequences.

Examination of a phylogenetic tree for genotypes 1–4 shows that genotype 3 can be split into 3 clades, with one composed of rabbit HEV sequences and two of human/swine clades designated 3.1 and 3.2 (Fig. 5). The genotype 3 subtypes included in each clade can be found in Table S1. All but one genotype 4 sequence originated either from China or Japan. Following this observations, the genotype 3 sequences were divided into 3.1 and 3.2 clades and the genotype 4 sequences into Chinese and Japanese sequences to determine whether each of these clades had its own unique demographic history. It is interesting to note that, although 87.5% of the clade 3.1 variants were from Asia and 60% of the clade 3.2 variants were from Europe (Table S1), these clades were found to have similar histories (Fig. 6). Although both clades experienced increase and decrease in effective population size over the last 60 years the population size shift was more dramatic in 3.1 (Fig. 6a) than in 3.2 (Fig. 6b). The genotype 4 country-specific variants showed different patterns (Fig. 7). The HEV population circulating in China (Fig. 7a) increased in effective size from about 1890 to 1940 and remains constant to the present. The Japanese HEV variants (Fig. 7b) maintained a constant population size until ∼1990 when it started to decrease rapidly, suggesting different population dynamics for the genotype 4 lineages in China and Japan.

## Discussion

Genotypes 3 and 4 are enzootic and zoonotic and can infect a number of species [3], [4], [17], [18], [32]. Adaptation of each strain to a range of hosts [34] should lead to a greater demand for genetic changes in the genome. Indeed, genotypes 3 and 4 were found here to have the higher fraction of variable sites in ORF1 and ORF2.N, even though most polymorphic sites were negatively selected. A larger fraction of invariant sites in genotype 1 leads to reduced intra-genotype heterogeneity and most probably reflects adaptation to a single host. While geographic constraints and isolation should lead to genetic diversity the lower geographic diversity with greater number of hosts and higher genetic diversity seen in genotype 4 versus genotype 1 may reflect a higher genetic diversity for adaption to a greater number of hosts in genotype 4 [1], [2], [4].

Until recently, HEV was only known to infect humans, swine, deer, boar and avians [3], [4], [9], [17], [18], [28]. These observations suggested a simple model of evolution, in which mammalian HEV infected humans and artiodactyls (swine, deer and boars). The discovery of HEV variants in rabbits [17] and rats [35] indicates, however, a more complex model of evolution for HEV. Using HEV sequences from avian and rat to root genotypes 1–4, we showed that HEV lineages can be split into two clades, genotype 1/2 and genotype 3/4, or anthropotropic and enzootic HEV genotypes, although this result should be viewed with some caution because of the single genotype 2 sequence used in the calculation. Divergence time analysis shows that the ancestor of genotypes 1–4 split into anthropotropic and enzootic genotypes about 536 to 1344 years ago (range; 536 to 865 years ago, ORF2.N and 816 to 1344 years ago, ORF1). It is not possible to order this split to determine whether the ancestor was anthropotropic or enzootic, although the rooting of these sequences with a sequence from HEVinfecting a rat suggests the ancestor was enzootic. The split which led to human and swine variants occurred at about the same time. The TMRCA for the avian/mammalian HEV ancestor and for the rat/[human/swine] HEV ancestor suggests that the most ancient split resulted in avian and mammalian variants. The sequence from HEV infecting a rat suggests that mammalian HEV has adapted to different mammalian species over time, and there may be mammalian intermediates leading from the avian/mammalian ancestor to humans and swine, which have not been discovered. It should be noted, however, that addition of novel HEV sequences may affect estimates for TMRCA, if these sequences would affect genetic heterogeneity of HEV genotypes.

There is a disparity between the estimated TMRCA values for modern HEV and the suggested evolutionary history. Intuitively, the estimated TMRCA values should be greater than these estimates. The disparity in ORF1 may, in part, be caused by the removal of the polyproline region, although its evolution cannot be accurately estimated by the methods used. A recent analysis of mitochondrial genomes shows that calculated divergence times were two to six-fold shorter than the true dates [36]. This effect may also hold for viral RNAs. Such apparent underestimate in viral TMRCA has been recently discussed [27], [28], [37]. It was suggested that underestimates of viral divergence times may be caused by limitations of the models to adequately reflect evolutionary events. It is possible that virus-host cospeciation may have resulted in lower substitution rates over the long run. It is also possible that viruses have histories dating back tens of thousands to millions of years but early members have gone extinct and been replaced by the modern variants; thus, divergence times only estimate times to the appearance of these more modern variants. The time for origination of more extensive contacts between humans as well as between humans and swine also suggests a more ancient TMRCA for the appearance of HEV genotypes 1–4 as swine were domesticated about 11,000 years ago [38] and urbanization started about 7,000 years ago [39]. The most direct way to resolve this issue is to obtain viral RNA from ancient humans or animals infected with HEV as has been done recently for other pathogens [40], [41] and to create a more diverse sampling of HEV sequences from around the world.

A further examination of the individual genotypes using ORF2.N sequences shows that each exhibits its own distinctive population dynamic. The effective number of infections associated with the HEV genotype 1 increased within the last 35 years (∼1970) and has been stable for the last twenty years (Fig. 2). Genotypes 3 and 4 showed increases around 1940 to 1945 followed by decreases around 1990 (Figs. 3 and 4). This increase in population size coincided with World War II and may have resulted from the movement of some human populations from urban settings to more rural settings [14] or more lax sanitation procedures at that time. A potential confounding factor to this conjecture is lowered consumption of pork in Japan during and immediately after the war. However, the effective population size for HEV is related to the rate of exposure rather than to meat consumption. Indeed Tanaka *et al*. showed that the effective population of HEV increases throughout the war. They believe the increase in HEV was related to the importation of infected pigs from England in 1900 followed by the growth of the population of imported pigs. Transmission to susceptible native swine should further increase the effective population. The reason for the decrease in the effective populations of genotypes 3 and 4 starting about 1990 is unknown. This decline suggests that the emergence of HEV seen in recent years may be due to greater awareness of the HEV health problem in the world and improved diagnostics rather than an actual expansion of the HEV viral population.

Genotype 3 can be split into three clades; namely, 3.1, 3.2 and rabbit strains. The removal of the rabbit sequences does not modify the skyline plot when all other genotype 3 sequences were analyzed. The 3.1 and 3.2 clades show similar skyline plots with similar trends, although the 3.2 clade did not show the same levels of population increase and decrease as 3.1. This suggests that both genotype 3 clades have experienced similar evolutionary history even though they represent different geographic distributions, with 3.1 being predominantly represented with HEV variants detected in Japan and 3.2 in Europe (Table S1).

Almost all the genotype 4 sequences used in the present study were recovered from HEV strains circulating in China or Japan. Thus, the evolutionary history of genotype 4 described here is the history of this genotype in these 2 countries. When the genotype 4 sequences were split into Chinese and Japanese sequences, different dynamics were observed. The Chinese sequences went through an increase in effective population over a period of about 55 years starting around 1890 to plateau over the last 65 years (Fig. 7). The Japanese sequences had a relatively constant effective population from 1900 to about 1990 and then decreased in size. These findings suggest significant differences in evolutionary processes acting in these two countries.

It is interesting to note that all 3 HEV genotypes, 1, 3 and 4, studied here experienced a population expansion. However, only genotypes 1 and 3 showed a rapid increase in the population size over 5–15 years (Figs. 2 and 3), with genotype 1 rapidly expanding in late 1970, and genotype 3 in the middle of the 20^th^ century. Genotype 4, however, exhibited a slow increase in the population size over the first half of the 20^th^ century (Fig. 4). A similar slow increase from 1880 to the middle of the 20^th^ century was observed for genotype 3 (Fig. 3). A dramatic decline in the population size for genotype 3 worldwide and genotype 4 in Japan over the last 15 years suggests a significant interruption in transmissions of these viral lineages, which could be associated with reduction in exposure. However, genotype 4 in China and genotype 1 do not show signs of decline, suggesting no dramatic changes in epidemiological processes acting on these lineages over the last 20–30 years. The country-specific HEV evolutionary history observed in this study most probably reflects temporal variations in rates of transmission and/or exposure for HEV strains of the same genotype circulating in different geographic regions. Analysis of population dynamics allows for distinguishing between the rate of detection and variation in the infected population size and presents an important tool for the identification of emerging diseases.

The dataset used in this study has several limitations that may potentially contribute to bias in the calculations presented here. First, the distribution of genotypic sequences is skewed primarily toward genotype 3 and 4 sequences, with only a single genotype 2 sequence being available. Second, the genotype 3 and 4 sequences are obtained mostly from HEV strains circulating in China and Japan, with only a limited number of sequences obtained from other parts of the world. Such geographic sequence representation could bias our analysis toward a representation of the history of HEV mainly in China and Japan. However, as was shown above, the skyline plots share a significant similarity between clades 3.1 and 3.2 of genotype 3, while sequences from these 2 clades have a noticeable difference in geographic origin. Nevertheless, the use of additional genotype 3 and 4 sequences, when available, outside China and Japan should help more accurate define the evolutionary history of HEV and most likely yield longer times to the most recent common ancestor for genotypes 3 and 4 and the genotype 3/4 ancestor. Third, there is no a known history or fossil records that can be used to confirm our analysis. The history of domestication of swine and the process of human urbanization; however, suggest that the Bayesian analysis presented here may be an underestimation of the evolutionary time for HEV.

## Supporting Information

Table S1 A list of the sequences used in the analyses. Sequences are listed by their GenBank accession numbers. Additional information includes the strain designation, genotype, date of collection, country of origin and species infected. The columns labeled ORF1, ORF2 and ORF3 are used to show which sequences were used to examine the substitution rate and evolutionary history of each ORF. The O in these columns indicates that the marked sequences were used in an earlier analysis with less data. The X indicates that these sequences with the sequences marked with an O were used in our final analysis. The years of collection enclosed in brackets are estimated dates of collection based on a reading of the papers published about these sequences. Genotype 3 sequences are further characterized by their subtypes. Figure 5 and the analysis in Figure 6 divide genotype 3 sequences into two clades. The sequences included in the 3.1 clade are those in bold text. The 3.2 clade sequences are those marked with italic text.
(0.20 MB DOC)

Table S2 Sequence modifications. The sequences used in this paper had to be modified for the reasons listed in Materials and Methods. The start and stop positions for the bases used in this study are listed in this table. These positions are based on the nucleotide numbering of reference sequences; M80581 (genotype 1), M74506 (genotype 2), AB248520 (genotype 3) and AB220979 (genotype 4). Part A shows the stop and stop positions used for ORF1 with the 5′ end listing positions used before the polyproline region and the 3′ end for the positions used after the polyproline region. Part B shows the start and stop positions for ORF2. ORF2 was further split into overlap (ORF2.O) and non-overlap (ORF2.N) regions as noted in Materials and Methods. Part C shows the start and stop positions for ORF3.
(0.05 MB DOC)

Table S3 Calculated TMRCA values for ORF1 and ORF2.N for all models tested. The values for the time to the most recent common ancestor were calculated using BEAST using the expanded sequence data set (See table S1). Part A lists values derived from the ORF2.N sequences. Part B lists values derived from the ORF1 sequences. See Table S2 and Materials and Methods for all modifications made to these sequences. These values are calculated using strict and relaxed clocks, an uncorrelated lognormal relaxed clock and an uncorrelated exponential relaxed clock with coalescent constant size (const), exponential (expon) and expansion (expan) growth tree priors. The mean time and limits of the 95% highest posterior probability density are shown for each ancestor. The genotypes column shows which genotypes belong to each ancestor. Where no values are listed for the genotype 3 & 4 ancestor for the uncorrelated exponential relaxed clock this is because genotypes 3 and 4 do not share a common ancestor in this model. The bold text values denote models for which some variables have an ESS below 200.
(0.09 MB DOC)
