# Supplementary material for: Evolutionary History and Population Dynamics of Hepatitis E Virus
Source: PLoS One. 2010 Dec 17;5(12):e14376. doi: 10.1371/journal.pone.0014376 (PMC3006657; doi:10.1371/journal.pone.0014376)
Supplement: Table S1 — A list of the sequences used in the analyses. Sequences are listed by their GenBank accession numbers. Additional information includes the strain designation, genotype, date of collection, country of origin and species infected. The columns labeled ORF1, ORF2 and ORF3 are used to show which sequences were used to examine the substitution rate and evolutionary history of each ORF. The O in these columns indicates that the marked sequences were used in an earlier analysis with less data. The X indicates that these sequences with the sequences marked with an O were used in our final analysis. The years of collection enclosed in brackets are estimated dates of collection based on a reading of the papers published about these sequences. Genotype 3 sequences are further characterized by their subtypes. Figure 5 and the analysis in Figure 6 divide genotype 3 sequences into two clades. The sequences included in the 3.1 clade are those in bold text. The 3.2 clade sequences are those marked with italic text. (0.20 MB DOC) [file pone.0014376.s001.doc]

|  |  | genotype | ORF1 | ORF2 | ORF3 | collected | |  | geographic location | |  |  |
| --- | --- | --- | --- | --- | --- | --- | --- | --- | --- | --- | --- | --- |
| ID | strain |  |  |  |  | year | mo | day | country | region | subregion | species |
| AB073912 | swJ570 | **3b** | O | O |  | 2000 | 9 | 11 | Japan |  |  | swine |
| AB074915 | JAK-Sai | 4 | O | O |  | 1994 | 7 | 12 | Japan | Saitama |  | homo sapiens |
| AB074917 | JKK-Sap | 4 | O | X | X | 2000 |  |  | Japan | Sapporo |  | homo sapiens |
| AB074918 | JKN-Sap | **3a** | O | O |  | 2001 | 7 | 6 | Japan | Sapporo |  | homo sapiens |
| AB074920 | JMY-Haw | **3a** | O | O |  | 2001 | 4 | 26 | US | Hawaii |  | homo sapiens |
| AB080575 | HE-JI4 | 4 |  | X | X | 2000 |  |  | Japan |  |  | homo sapiens |
| AB089824 | HE-JA10 | **3a** | X | X | X | 1993 |  |  | Japan | Tokyo |  | homo sapiens |
| AB091394 | JJT-Kan | **3b** |  | O |  | 1998 | 11 | 21 | Japan | Kanagawa |  | homo sapiens |
| AB091395 | JSN-Sap-FH | 4 |  | O |  | 2002 | 3 | 21 | Japan | Hokkaido |  | homo sapiens |
| AB097811 | swJ13-1 | 4 | X | X | X | 2002 |  |  | Japan | Hokkaido |  | swine |
| AB097812 | HE-JA1 | 4 | X | X | X | 1997 |  |  | Japan | Hokkaido |  | homo sapiens |
| AB099347 | HE-JK4 | 4 | X | X | X | 2002 |  |  | Japan | Tochigi |  | homo sapiens |
| AB108537 | CCC220 | 4 | X | X | X | 2000 |  |  | China | Changchun |  | homo sapiens |
| AB161717 | JSM-Sap95 | 4 | X | X | X | 1995 |  |  | Japan | Hokkaido | Sapporo | homo sapiens |
| AB161718 | JTS-Sap02 | 4 | X | X | X | 2002 |  |  | Japan | Hokkaido | Sapporo | homo sapiens |
| AB161719 | JYW-Sap02 | 4 | X | X | X | 2002 |  |  | Japan | Hokkaido | Sapporo | homo sapiens |
| AB189070 | JBOAR1-Hyo04 | **3b** | X | X | X | 2004 | 4 | 8 | Japan | Hyogo | Taka-gun | wild boar |
| AB189071 | JDEER-Hyo03L | **3b** | X | O | X | 2003 | 2 | 22 | Japan | Hyogo | Taka-gun | deer |
| AB189072 | JMO-Hyo03L | **3b** | X | X | X | 2003 | 4 | 29 | Japan | Hyogo | Kasai | homo sapiens |
| AB189073 | JSO-Hyo03L | **3b** | X | X | X | 2003 | 4 | 29 | Japan | Hyogo | Kasai | homo sapiens |
| AB189074 | JTH-Hyo03L | **3b** | X | X | X | 2003 | 4 | 29 | Japan | Hyogo | Kasai | homo sapiens |
| AB189075 | JYO-Hyo03L | **3b** | X | O | X | 2003 | 4 | 29 | Japan | Hyogo | Kasai | homo sapiens |
| AB193176 | JSF-Tot03C | 4 | X | X | X | 2003 | 3 | 12 | Japan | Tottori |  | homo sapiens |
| AB193177 | JYN-Sap01C | 4 | X | X | X | 2001 | 12 | 23 | Japan | Hokkaido | Sapporo | homo sapiens |
| AB193178 | JYN-Nii02L | 4 | X | X | X | 2002 | 4 | 30 | Japan | Niigata |  | homo sapiens |
| AB197673 | JKO-ChiSai98C | 4 | X | X | X | 1998 | 10 | 17 | China | Xian |  | homo sapiens |
| AB197674 | JYI-ChiSai01C | 4 | O |  |  | 2001 | 4 | 12 | China | Shanghai |  | homo sapiens |
| AB200239 | JSN-Sap-FH02C | 4 |  | X | X | 2002 | 3 | 21 | Japan | Hokkaido | Sapporo | homo sapiens |
| AB220971 | He-JF3 | 4 | O | O |  | 1998 | 6 | 19 | Japan | Mito |  | homo sapiens |
| AB220972 | HE-JF4 | 4 |  | O |  | 2002 | 10 | 2 | Japan | Hokkaido |  | homo sapiens |
| AB220973 | HE-JF5 | 4 | O | O |  | 2002 | 12 | 2 | Japan | Hokkaido |  | homo sapiens |
| AB220975 | He-JA19 | 4 | O | O |  | 2002 | 12 | 24 | Japan | Hokkaido |  | homo sapiens |
| AB220979 | HE-JA41 | 4 |  | O |  | 2004 | 8 | 17 | Japan | Hokkaido |  | homo sapiens |
| AB222182 | wbJSG1 | **3b** |  | O |  | 2003 | 12 | 19 | Japan | Saga |  | wild boar |
| AB222183 | wbJTS1 | **3b** | X | X | X | 2004 | 12 | 9 | Japan | Tokushima |  | wild boar |
| AB222184 | wbJYG1 | **3b** |  | O |  | 2005 | 2 | 6 | Japan | Yamaguchi |  | wild boar |
| AB236320 | JMNG-Oki02C | **3b** | O | O | X | 2002 |  |  | Japan | Okinawa |  | mongoose |
| AB248520 | HE-JA04-1911 | *3b* | X | X | X | 2004 |  |  | Japan | Mie |  | homo sapiens |
| AB290312 | swMN06-A1288 | **3c** | X | X | X | 2006 |  |  | Mongolia |  |  | swine |
| AB290313 | swMN06-C1056 | *3f* | X | X | X | 2006 |  |  | Mongolia |  |  | swine |
| AB291951 | JIO-Sai97L | **3b** | O | O |  | 1997 | 3 | 29 | Japan | Saitama |  | homo sapiens |
| AB291952 | JIY-Tot05L | **3b** | O | O |  | 2005 | 6 | 24 | Japan | Tottori |  | homo sapiens |
| AB291953 | JSO-Oki05L | **3b** | O | O |  | 2005 | 7 | 31 | Japan | Okinawa |  | homo sapiens |
| AB291954 | JSS-Oka04L | **3b** | O | O |  | 2004 | 12 | 24 | Japan | Okayama |  | homo sapiens |
| AB291955 | JSW-Kyo-FH06L | **3b** | O | O |  | 2006 | 9 | 5 | Japan | Kyoto |  | homo sapiens |
| AB291956 | JYM-Tot04L | **3b** | O | O |  | 2004 | 1 | 19 | Japan | Tottori |  | homo sapiens |
| AB291957 | JYU-Oki04L | **3b** | O | O |  | 2004 | 5 | 18 | Japan | Okinawa |  | homo sapiens |
| AB291958 | JNH-Ehi04L | *3e* | O | O |  | 2004 | 6 | 17 | Japan | Ehime |  | homo sapiens |
| AB291959 | JTC-Kit-FH04L | 4 |  |  |  | 2004 | 9 | 24 | Japan | Hokkaido | Kitami | homo sapiens |
| AB291960 | JTK-Kag06C | **3b** | O | O |  | 2006 | 3 | 23 | Japan | Kagawa |  | homo sapiens |
| AB291961 | JMH-Osa04C | *3f* | O | O |  | 2004 | 3 | 10 | Japan | Osaka |  | homo sapiens |
| AB291962 | JHK-Toy04C | **3b** | O | O |  | 2004 | 6 | 16 | Japan | Toyama |  | homo sapiens |
| AB291963 | JRM-Toy05C | **3b** | O | O |  | 2005 | 11 | 28 | Japan | Toyama |  | homo sapiens |
| AB291964 | JYK-Tok03C | 4 |  | O |  | 2003 | 6 | 9 | Japan | Tokyo |  | homo sapiens |
| AB291965 | HRC-HE14C | 4 |  | O |  | 2004 | 9 | 20 | Japan | Hokkaido | Kitami | homo sapiens |
| AB291966 | JST-KitAsa04C | 4 |  |  |  | 2004 | 10 | 12 | Japan | Hokkaido | Asahikawa | homo sapiens |
| AB291967 | JKO-Aba-FH06C | 4 | O | O |  | 2006 | 3 | 10 | Japan | Hokkaido | Abashiri | homo sapiens |
| AB291968 | JMM-Aba06C | 4 | O | O |  | 2006 | 3 | 9 | Japan | Hokkaido | Abashiri | homo sapiens |
| AB301710 | JE03-1760F | **3b** | X | X | X | 2003 |  |  | Japan |  |  | homo sapiens |
| AB369687 | E116-YKH98C | 3f |  |  |  | 1998 |  |  | Thailand |  |  | homo sapiens |
| AB369688 | E087-SAP04C | 4 | X | X | X | 2004 |  |  | China | Shanghai |  | homo sapiens |
| AB369689 | E088-STM04C | **3a** | X | X | X | 2004 |  |  | Japan | Saitama |  | homo sapiens |
| AB369690 | E067-SIJ05C | 4 | X | X | X | 2005 |  |  | China | Shanghai |  | homo sapiens |
| AB369691 | E097-OSA05C | 3b | O |  |  | 2005 |  |  | Japan | Osaka |  | homo sapiens |
| AB437316 | JE03-1760F | **3b** | O | O |  | 2003 |  |  | Japan |  |  | homo sapiens |
| AB437317 | JE03-1760F | **3b** | O | O |  | 2003 |  |  | Japan |  |  | homo sapiens |
| AB437318 | JE03-17 | **3b** | O | O |  | 2003 |  |  | Japan |  |  | homo sapiens |
| AB437319 | JE03-1760F | **3b** |  | O |  | 2003 |  |  | Japan |  |  | homo sapiens |
| AB443623 | JIO-swJ19-1 | 3b | O |  |  | 2002 |  |  | Japan | Miyazaki |  | swine |
| AB443624 | JIO-swJ19-2 | 3b | O |  |  | 2002 |  |  | Japan | Miyazaki |  | swine |
| AB443625 | JIO-swJ19-5 | 3b | O |  |  | 2002 |  |  | Japan | Miyazaki |  | swine |
| AB443626 | JIO-swJ19-7 | 3b | O |  |  | 2002 |  |  | Japan | Miyazaki |  | swine |
| AB443627 | JIO-swJ19-8 | **3b** | O | O |  | 2002 |  |  | Japan | Miyazaki |  | swine |
| AF051350 | M7 (80-Algeria) | 1 |  |  | X | 1980 |  |  | Algeria |  |  | homo sapiens |
| AF051351 | 93-Egypt | 1 |  | X | X | 1993 |  |  | Egypt | Cairo |  | homo sapiens |
| AF051352 | 94-Egypt | 1 |  | X | X | 1994 |  |  | Egypt | Cairo |  | homo sapiens |
| AF051830 | TK-15/92 | 1 | X | X | X | 1992 |  |  | Nepal | Kathmandu |  | homo sapiens |
| AF060668 | US-1 | 3a | X | X | X | 1995 |  |  | USA |  |  | swine |
| AF065061 | HEV-Morocco | 1 |  | X | X | 1994 |  |  | Morocco |  |  | homo sapiens |
| AF076239 |  | 1 | X | X | X | 1990 |  |  | India | Hyderabad |  | homo sapiens |
| AF082843 | USA-S | **3a** | X | X | X | 1996 |  |  | USA |  |  | swine |
| AF124406 | AKL-90 | 1 |  |  | X | 1990 |  |  | India | Akluj |  | homo sapiens |
| AF124407 | AKL-90 | 1 |  | X |  | 1990 |  |  | India | Akluj |  | homo sapiens |
| AF185822 | Abb-2B | 1 | X | X | X | 1988 |  |  | Pakistan | Abbottabad |  | homo sapiens |
| AF459438 | Yam-67 | 1 | O | O |  | 1989 |  |  | India | Yamuna Nagar | Haryana | homo sapiens |
| AY204877 | T3 | 1 | X | X | X | 1983 |  |  | Chad |  |  | homo sapiens |
| AY230202 |  | 1 | O | O |  | 1994 |  |  | Morocco |  |  | homo sapiens |
| AY575857 | pSHEV-1 | **3a** | O | O |  | 1996 |  |  | US | Illinois |  | swine |
| D10330 | HEVNE8L | 1 | X | X | X | 1989 |  |  | Myanmar | Burma |  | homo sapiens |
| D11092 | China-A | 1 | X | X | X | 1987 |  |  | China |  |  | homo sapiens |
| D11093 | China-D (Uigh179) | 1 | X | X | X | 1987 |  |  | China | Xinjiang |  | homo sapiens |
| DQ079629 | FF031605 | **3b** |  | X |  | 2005 |  |  | Japan | Iizuka |  | homo sapiens |
| DQ079630 | WB011905 | 4 |  | O |  | 2004 |  |  | Japan |  |  | wild boar |
| DQ459342 |  | 1 | X | X | X | 2000 |  |  | India | western region |  | homo sapiens |
| EU375463 | Thai-swHEV07 | *3f* | O | O |  | 2006 | 12 | 26 | Thailand |  |  | swine |
| EU723512 | SW626 | *3f* | O | O |  | 2005 |  |  | Spain |  |  | swine |
| EU723513 | SW627 | *3f* | O | O |  | 2005 |  |  | Spain |  |  | swine |
| EU723514 | SWP6 | *3f* | O | O |  | 2006 |  |  | Spain |  |  | swine |
| EU723515 | SWP7 | *3f* | O | O |  | 2006 |  |  | Spain |  |  | swine |
| EU723516 | SWP8 | *3f* | O | O |  | 2006 |  |  | Spain |  |  | swine |
| FJ426403 | swKOR-1 | **3a** | O | O |  | 2007 |  |  | South Korea |  |  | swine |
| FJ426404 | swKOR-2 | **3a** | O | O |  | 2007 |  |  | South Korea |  |  | swine |
| FJ457024 | HEV-H | 1 | O | O |  | 2005 |  |  | India |  |  | homo sapiens |
| FJ527832 | SAAS-JDY5 | **3b** | O | O |  | 2007 | 1 | 7 | China | Shanghai |  | swine |
| FJ610232 | swCH189 | 4 | O | O |  | 2008 | 3 | 18 | China | Gansu |  | swine |
| FJ705359 | wbGER27 | **3c** | O | O |  | 2006 |  |  | Germany |  |  | wild boar |
| FJ906895 |  | 3 | O | O |  | [2008] |  |  | China |  |  | rabbit |
| FJ906896 |  | 3 | O | O |  | [2008] |  |  | China |  |  | rabbit |
| FJ956757 | HEV_RKI | *3f* | O | O |  | 2005 | 7 | 7 | Germany |  |  | homo sapiens |
| FJ998008 | BB02 | **3c** |  | O |  | 2007 | 11 | 25 | Germany | Krausnick | Brandenburg | wild boar |
| FJ998015 | SA21 | *3e* |  | O |  | 2007 | 12 | 1 | Germany | Welzow-Proschim | Brandenburg/Saxony | wild boar |
| GU188851 | WH09 | 4 | O | O |  | 2009 | 3 | 27 | China |  |  | swine |
| GU206559 | bjsw1 | 4 | O | O |  | 2008 | 2 |  | China |  |  | swine |
| L25595 | K52-87 | 1 |  | X | X | 1987 |  |  | China | Xinjiang |  | homo sapiens |
| M73218 | Burma | 1 | X | X | X | 1982 |  |  | Myanmar |  |  | homo sapiens |
| M74506 | MEX-14 | 2 | X | X | X | 1986 |  |  | Mexico | Telixtac |  | homo sapiens |
| M80581 | Sar-55 | 1 | X | X | X | 1987 |  |  | Pakistan | Sargodha |  | homo sapiens |
| M94177 | HeBei | 1 | X | X | X | 1987 |  |  | China | Xinjiang |  | homo sapiens |
| X98292 | hev037 | 1 | X | X | X | 1992 |  |  | India |  |  | homo sapiens |
| X99441 |  | 1 | X | X | X | 1993 |  |  | India | Madras |  | homo sapiens |
|  |  |  |  |  |  |  |  |  |  |  |  |  |
| Outgroup |  |  |  |  |  |  |  |  |  |  |  |  |
| AY535004 |  |  |  | O |  | [2001] |  |  | US |  |  | chicken |
| EF206691 |  |  |  | O |  | [2001] |  |  | US |  |  | chicken |
| GQ504009 |  |  |  | X |  | 2008 |  |  | Germany | Hamburg |  | rat |
