# Supplementary material for: Evolutionary History and Population Dynamics of Hepatitis E Virus
Source: PLoS One. 2010 Dec 17;5(12):e14376. doi: 10.1371/journal.pone.0014376 (PMC3006657; doi:10.1371/journal.pone.0014376)
Supplement: Table S2 — Sequence modifications. The sequences used in this paper had to be modified for the reasons listed in Materials and Methods. The start and stop positions for the bases used in this study are listed in this table. These positions are based on the nucleotide numbering of reference sequences; M80581 (genotype 1), M74506 (genotype 2), AB248520 (genotype 3) and AB220979 (genotype 4). Part A shows the stop and stop positions used for ORF1 with the 5′ end listing positions used before the polyproline region and the 3′ end for the positions used after the polyproline region. Part B shows the start and stop positions for ORF2. ORF2 was further split into overlap (ORF2.O) and non-overlap (ORF2.N) regions as noted in Materials and Methods. Part C shows the start and stop positions for ORF3. (0.05 MB DOC) [file pone.0014376.s002.doc]

| A | 5’ end ORF1 | |  | 3’ end ORF1 |  |
| --- | --- | --- | --- | --- | --- |
| genotype | start | stop |  | start | stop |
| 1 | 22 | 2151 |  | 2323 | 5079 |
| 2 | 20 | 2157 |  | 2323 | 5079 |
| 3 | 50 | 2177 |  | 2423 | 5179 |
| 4 | 50 | 2179 |  | 2393 | 5149 |
|  |  |  |  |  |  |
| B | ORF2 | |  |  |  |
| genotype | start | stop |  |  |  |
| 1 | 5117 | 7096 |  |  |  |
| 2 | 5117 | 7096 |  |  |  |
| 3 | 5214 | 7196 |  |  |  |
| 4 | 5188 | 7170 |  |  |  |
|  |  |  |  |  |  |
| C | ORF3 | |  |  |  |
| genotype | start | stop |  |  |  |
| 1 | 5103 | 5447 |  |  |  |
| 2 | 5103 | 5447 |  |  |  |
| 3 | 5203 | 5544 |  |  |  |
| 4 | 5174 | 5518 |  |  |  |
