# Supplementary material for: Evolutionary History and Population Dynamics of Hepatitis E Virus
Source: PLoS One. 2010 Dec 17;5(12):e14376. doi: 10.1371/journal.pone.0014376 (PMC3006657; doi:10.1371/journal.pone.0014376)
Supplement: Table S3 — Calculated TMRCA values for ORF1 and ORF2.N for all models tested. The values for the time to the most recent common ancestor were calculated using BEAST using the expanded sequence data set (See table S1). Part A lists values derived from the ORF2.N sequences. Part B lists values derived from the ORF1 sequences. See Table S2 and Materials and Methods for all modifications made to these sequences. These values are calculated using strict and relaxed clocks, an uncorrelated lognormal relaxed clock and an uncorrelated exponential relaxed clock with coalescent constant size (const), exponential (expon) and expansion (expan) growth tree priors. The mean time and limits of the 95% highest posterior probability density are shown for each ancestor. The genotypes column shows which genotypes belong to each ancestor. Where no values are listed for the genotype 3 & 4 ancestor for the uncorrelated exponential relaxed clock this is because genotypes 3 and 4 do not share a common ancestor in this model. The bold text values denote models for which some variables have an ESS below 200. (0.09 MB DOC) [file pone.0014376.s003.doc]

| 1. ORF2.N | | | | Relaxed Uncorrelated Clock | | | |
| --- | --- | --- | --- | --- | --- | --- | --- |
|  | | Strict Clock | | Lognormal | | Exponential | |
| Genotypes | Prior | Mean | HPD | Mean | HPD | Mean | HPD |
| 1, 2, 3 & 4 | Expon | 521.99 | 449.78-597.49 | 523.99 | 418.76-638.76 | 1096.25 | 442.99-2047.67 |
| 1, 2, 3 & 4 | Expan | 531.00 | 457.44-608.63 | 544.00 | 429.18-665.37 | 781.51 | 331.70-1400.10 |
| 1, 2, 3 & 4 | Const | 536.49 | 462.04-614.08 | 556.20 | 437.69-686.09 | 864.78 | 434.16-1455.83 |
| 3 & 4 | Expon | 407.36 | 343.20-470.95 | 410.50 | 330.88-510.36 |  |  |
| 3 & 4 | Expan | 413.85 | 348.56-478.29 | 418.93 | 328.88-511.38 |  |  |
| 3 & 4 | Const | 416.82 | 354.04-485.58 | 423.74 | 336.84-519.03 |  |  |
| 1 & 2 | Expon | 442.85 | 367.02-524.91 | 410.81 | 283.97-535.29 | 410.23 | 141.47-756.13 |
| 1 & 2 | Expan | 453.01 | 374.11-536.87 | 436.44 | 302.40-577.63 | 329.92 | 150.79-577.30 |
| 1 & 2 | Const | 457.66 | 375.23-540.64 | 441.44 | 309.47-588.95 | 367.18 | 154.89-665.78 |
| 3 | Expon | 261.51 | 229.23-293.49 | 272.72 | 227.63-320.13 | 452.70 | 228.65-766.22 |
| 3 | Expan | 263.54 | 232.95-297.77 | 276.15 | 230.54-329.26 | 376.92 | 206.55-609.98 |
| 3 | Const | 264.54 | 233.40-298.70 | 277.11 | 229.66-326.38 | 421.26 | 213.42-678.09 |
| 4 | Expon | 130.16 | 118.53-141.48 | 146.06 | 123.31-169.73 | 261.49 | 114.01-468.11 |
| 4 | Expan | 130.11 | 118.16-141.52 | 145.89 | 123.60-170.46 | 236.12 | 109.80-391.65 |
| 4 | Const | 130.73 | 119.56-143.00 | 146.82 | 124.01-171.23 | 240.82 | 107.36-415.90 |
| 1 | Expon | 92.86 | 84.25-102.46 | 93.96 | 80.62-109.21 | 149.28 | 73.22-260.79 |
| 1 | Expan | 92.84 | 83.95-102.52 | 94.23 | 80.53-110.60 | 140.78 | 71.30-229.91 |
| 1 | Const | 93.10 | 83.76-102.52 | 94.40 | 79.68-109.59 | 146.44 | 68.98-246.24 |
|  |  |  |  |  |  |  |  |
| 1. ORF1 | |  |  | Relaxed Uncorrelated Clock | | | |
|  |  | Strict Clock | | Lognormal | | Exponential | |
| Genotypes | Prior | Mean | HPD | Mean | HPD | Mean | HPD |
| 1, 2, 3 & 4 | Expon | 814.03 | 756.31-870.37 | 819.77 | 713.58-928.71 | **1188.94** | **478.95-2164.42** |
| 1, 2, 3 & 4 | Expan | 813.35 | 757.60-871.62 | 819.24 | 716.72-930.34 | **933.66** | **529.41-1496.31** |
| 1, 2, 3 & 4 | Const | 816.44 | 761.28-877.48 | 814.03 | 756.31-870.37 | **1343.53** | **488.50-2412.12** |
| 3 & 4 | Expon | 677.60 | 622.90-733.89 | 689.48 | 583.14-797.04 |  |  |
| 3 & 4 | Expan | 676.95 | 619.56-729.59 | 692.10 | 584.49-802.24 | **566.99** | **315.31-812.69** |
| 3 & 4 | Const | 679.23 | 621.66-733.37 | 677.60 | 622.90-733.89 | **614.87** | **298.80-983.28** |
| 1 & 2 | Expon | 652.84 | 592.61-719.15 | 643.62 | 499.44-795.12 | **420.41** | **198.15-765.71** |
| 1 & 2 | Expan | 653.33 | 589.71-714.26 | 641.26 | 496.77-795.17 | **505.93** | **220.22-864.51** |
| 1 & 2 | Const | 655.54 | 593.76-719.45 | 652.84 | 592.61-719.15 | **498.96** | **269.33-960.38** |
| 3 | Expon | 337.45 | 311.79-363.26 | 343.04 | 298.81-388.03 | **484.54** | **247.28-846.42** |
| 3 | Expan | 337.04 | 311.56-363.91 | 344.21 | 300.34-391.85 | **407.04** | **234.22-638.92** |
| 3 | Const | 337.68 | 311.89-364.43 | 337.45 | 311.79-363.26 | **431.81** | **205.00-699.57** |
| 4 | Expon | 146.35 | 138.70-153.81 | 148.05 | 133.87-162.72 | **237.57** | **94.10-402.30** |
| 4 | Expan | 146.22 | 138.58-154.04 | 147.13 | 133.30-161.37 | **254.11** | **111.05-446.02** |
| 4 | Const | 146.38 | 138.74-154.22 | 146.35 | 134.02-151.20 | **266.28** | **130.39-455.44** |
| 1 | Expon | 86.70 | 82.02-91.75 | 88.25 | 78.37-98.58 | **168.74** | **81.30-257.86** |
| 1 | Expan | 86.68 | 81.77-91.42 | 88.18 | 78.81-98.43 | **170.47** | **77.22-316.21** |
| 1 | Const | 86.73 | 81.80-91.65 | 86.70 | 82.002-91.75 | **198.89** | **87.24-408.47** |
